# Clinicopathological Predictors of Recurrence in Resected Stage IIB-IIIB Melanoma: The Prognostic Impact of Stage IIC in a Real-World Cohort

**DOI:** 10.3390/cancers18162694

**Published:** 2026-08-20

**Authors:** Icíar De La Fuente Domínguez, Pedro Sánchez Mauriño, Luis Pérez Bartivas, María Sánchez-Lopera, Rafael Sánchez Sánchez, Enrique Aranda Aguilar

**Affiliations:** 1 University of Córdoba, Maimonides Biomedical Research Institute of Córdoba (IMIBIC), Reina Sofía University Hospital, 14004 Córdoba, Spain; 2 Department of Medicine, Faculty of Medicine, University of Córdoba, 14071 Córdoba, Spain; 3 Medical Oncology Department, Reina Sofía University Hospital, 14004 Córdoba, Spain; 4 Anatomopathological Department, Reina Sofía University Hospital, 14004 Córdoba, Spain

**Keywords:** melanoma, stage IIC, sentinel lymph node, recurrence, prognosis, BRAF, adjuvant immunotherapy

## Abstract

**Background**: Patients with stage IIB–IIIB cutaneous melanoma remain at substantial risk of recurrence despite complete surgical resection. Accurate risk stratification is essential to optimize patient selection for adjuvant therapy and surveillance strategies. We aimed to evaluate the clinicopathological and molecular factors associated with recurrence in a real-world cohort of high-risk melanoma patients. **Methods**: We retrospectively analyzed 97 patients with stage IIB–IIIB cutaneous melanoma treated at a single tertiary referral center. Clinicopathological characteristics, molecular features, treatment-related variables, and sentinel lymph node (SLN) pathological characteristics were evaluated. Recurrence-free survival (RFS) was estimated using the Kaplan–Meier method. Independent prognostic factors were identified using multivariable Cox proportional hazards regression. **Results**: After a median follow-up of 88 months, 31 patients (32.0%) developed recurrence. Stage IIC showed the highest recurrence rate in the cohort (52.4%), compared with 25% in stage IIIA and 31.4% in stage IIIB. Consistently, in the multivariable analysis, compared with stage IIB, only stage IIC remained independently associated with worse recurrence-free survival (hazard ratio [HR] = 3.57, 95% confidence interval [CI]: 1.08–11.82; *p* = 0.037), whereas neither stage IIIA nor stage IIIB differed significantly from stage IIB. Female sex was independently associated with a lower risk of recurrence (HR 0.33, 95% CI 0.14–0.79; *p* = 0.012). Although BRAF V600E-mutated tumors showed higher recurrence rates and shorter recurrence-free survival in univariable analyses, BRAF V600E status was not independently associated with recurrence in the multivariable model (*p* = 0.250). Higher lymphoid infiltration density was associated with improved RFS in univariable analysis, although this finding was limited by substantial missing data and non-standardized pathological assessment. **Conclusions**: In this exploratory retrospective analysis, female sex and stage IIC were independently associated with RFS. These findings should be interpreted cautiously given the small sample size, limited number of recurrence events, and incomplete availability of some clinicopathological and molecular variables. The prognostic associations observed for BRAF V600E status and lymphoid infiltration, as well as the analyses according to adjuvant treatment, should be considered exploratory and hypothesis-generating. The unfavorable outcomes observed in stage IIC disease support growing evidence that this subgroup represents a biologically aggressive form of melanoma despite the absence of regional nodal involvement. Our findings do not challenge the validity of the AJCC staging system but suggest that clinically relevant heterogeneity may exist within established AJCC stage groups.

## Simple Summary

Patients with surgically resected stage IIB–IIIB melanoma remain at substantial risk of disease recurrence despite curative treatment. Identifying those at highest risk is increasingly important because adjuvant immunotherapy is now available for selected patients. In this real-world study, we evaluated the clinical, pathological and molecular factors associated with recurrence in 97 patients treated at a tertiary referral center. We found that female sex was associated with a lower risk of recurrence, whereas stage IIC melanoma showed an unfavorable recurrence pattern and was independently associated with shorter recurrence-free survival compared with stage IIB. However, the numerical recurrence rate observed in stage IIC was higher than that observed in stage IIIB, and this difference was not directly tested statistically. Therefore, these findings should not be interpreted as evidence that stage IIC has worse outcomes than stage IIIB. Rather, they suggest that clinically relevant heterogeneity may exist within established AJCC stage groups. Pathological features of sentinel lymph node metastases did not show significant prognostic associations in this cohort. These exploratory findings require confirmation in larger independent cohorts.

## 1. Introduction

Cutaneous melanoma is a malignant neoplasm characterized by high metastatic potential. Although it accounts for only a small proportion of skin cancer diagnoses, it is responsible for the majority of skin cancer deaths. Its incidence has steadily increased worldwide over recent decades, particularly among fair-skinned populations. According to recent estimates from the Spanish Network of Cancer Registries (REDECAN), approximately 8074 new cases of cutaneous melanoma are expected to be diagnosed in Spain in 2026, corresponding to an age-standardized incidence rate of 15.1 cases per 100,000 inhabitants [1]. Despite recent therapeutic advances, melanoma-related mortality remains close to two deaths per 100,000 inhabitants annually in Spain, while contemporary nationwide survival estimates remain limited, highlighting the importance of long-term real-world data [2,3].

Ultraviolet (UV) radiation exposure remains the principal environmental risk factor for melanoma development. Additional risk factors include a fair skin phenotype, a high number of melanocytic nevi, congenital nevi, personal history of melanoma, and a positive family history of the disease. Furthermore, germline susceptibility variants affect genes involved in cell-cycle regulation and pigmentation [4,5]. Melanoma comprises several histopathological subtypes with distinct biological behavior, including superficial spreading, nodular, lentigo maligna, acral lentiginous, desmoplastic, and amelanotic melanoma types [6]. Anatomical distribution also varies by sex: melanomas more commonly arise on the trunk and head and neck in men, whereas extremity involvement, particularly of the lower limbs, is more frequent in women [7,8].

Definitive diagnosis requires histopathological examination following complete excisional biopsy of the lesion. Current staging is based on the eighth edition of the American Joint Committee on Cancer (AJCC) TNM classification system which incorporates Breslow thickness, ulceration, regional lymph node involvement, and distant metastases [6]. Breslow thickness is the vertical measurement, in millimeters, of the invasive melanoma from the granular layer of the epidermis (or from the base of an ulcer, when present) to the deepest point of tumor invasion. It is a major histopathological prognostic factor because increasing tumor thickness is associated with a higher risk of recurrence and mortality. Ulceration and sentinel lymph node (SLN) status provide additional prognostic information [6,9].

The standard treatment for localized melanoma is wide local excision with the margins determined according to tumor thickness. Sentinel lymph node biopsy (SLNB) is recommended for patients with melanomas ≥ 0.8 mm in thickness or for thinner lesions presenting additional high-risk features because it provides accurate pathological staging and valuable prognostic information [10]. Following the publication of the MSLT-II [11] and DeCOG-SLT [12] trials, completion lymph node dissection is no longer routinely recommended after a positive SLN biopsy, as active nodal surveillance provides comparable melanoma-specific survival with substantially lower morbidity [11,12,13].

Although AJCC staging remains the cornerstone of prognostic assessment, increasing evidence suggests that substantial biological heterogeneity exists within individual stage groups. Specifically, stage IIC corresponds to T4bN0M0 disease, defined by a primary tumor > 4 mm in thickness with ulceration and no regional or distant metastases [6]. Patients with stage IIB and especially stage IIC melanoma may experience recurrence rates approaching, or in some cohorts numerically exceeding, those observed in selected stage III subgroups despite the absence of regional nodal metastases [14,15,16]. This observation has become particularly relevant following the publication of the KEYNOTE-716 and CheckMate 76K phase III trials, which demonstrated significant improvements in recurrence-free survival with adjuvant anti-PD-1 therapy in completely resected stage IIB–IIC melanoma, leading to major changes in international treatment guidelines [17,18].

Despite these therapeutic advances, conventional clinicopathological variables remain insufficient to accurately identify patients at highest risk of recurrence. Among the molecular alterations investigated in melanoma, BRAF mutations have attracted particular interest because of their potential association with recurrence risk, although their independent prognostic value in localized disease remains controversial [19,20]. Likewise, additional pathological characteristics of SLN metastases—including metastatic tumor burden, anatomical distribution within the node, and metastatic morphology—have been proposed as potential prognostic biomarkers. The Rotterdam criteria and the Dewar classification have demonstrated prognostic value in large multi-center studies, identifying subgroups of SLN-positive patients with significantly different survival outcomes and risks of non-sentinel node involvement [21,22]. However, these pathological parameters are not routinely incorporated into current prognostic models and their role in the era of modern adjuvant immunotherapy remains uncertain.

The identification of additional clinicopathological and molecular factors associated with recurrence could improve patient selection for adjuvant treatment and facilitate a more individualized approach to surveillance. Therefore, the aim of the present study was to evaluate the prognostic factors associated with recurrence in a real-world cohort of patients with completely resected stage IIB–IIIB cutaneous melanoma treated at a tertiary referral center.

## 2. Materials and Methods

### 2.1. Study Design and Patient Population

This retrospective, single-center cohort study included consecutive adult patients diagnosed with completely resected stage IIB–IIIB cutaneous melanoma at Reina Sofía University Hospital (Córdoba, Spain). Patients were managed according to institutional clinical practice and subsequently followed according to routine surveillance protocols.

Eligible patients were aged ≥ 18 years and had histologically confirmed AJCC 8th edition stage IIB, IIC, IIIA, or IIIB cutaneous melanoma with an Eastern Cooperative Oncology Group (ECOG) performance status of 0–2. Patients with mucosal or uveal melanoma were excluded.

Histological subtypes were classified as lower-risk (superficial spreading melanoma and other favorable variants) or higher-risk (nodular, acral lentiginous, and pure desmoplastic melanoma). Primary tumor location was categorized as trunk/head and neck or extremities.

An exploratory pathological analysis was performed in the subgroup of patients with a positive SLNB. Metastatic deposits were classified according to the Dewar topographic classification, distinguishing subcapsular, combined, parenchymal, multifocal and extensive types [21]. Sentinel node tumor burden was additionally assessed by measuring the largest diameter of the metastatic deposit and categorized according to the Rotterdam criteria (≤1 mm, >1–5 mm, and >5 mm) [22]. In patients with multifocal nodal involvement, the largest metastatic focus was recorded. Metastatic morphology was classified as aggregates, isolated tumor cells, or nodular deposits based on the original pathology reports. Lymphoid infiltration was recorded from the original histopathological reports using the available ordinal categories (absent, mild, mild–moderate, moderate, or severe).

The study was conducted in accordance with the Declaration of Helsinki, and the protocol was approved by the Ethics Committee of Córdoba. Patient confidentiality was maintained through anonymized data collection and processing in accordance with Spanish data protection regulations, including Law 14/2007 on Biomedical Research and Law 41/2002 on Patient Autonomy.

### 2.2. Statistical Analysis

Statistical analyses were performed using IBM SPSS Statistics version 22.0 (IBM Corp., Armonk, NY, USA). RFS was defined as the time from primary surgery to disease recurrence or last follow-up for censored patients. Overall survival (OS) was calculated from the date of diagnosis to death from any cause or last follow-up. Survival probabilities were estimated using the Kaplan–Meier method and compared using the log-rank test.

Categorical variables were compared using Pearson’s Chi-square test or Fisher’s exact test as appropriate. Continuous variables were analyzed using Student’s *t*-test after assessment of normality and homogeneity of variance. Variables considered clinically relevant were prespecified as candidate covariates in the multivariable model. Hazard ratios (HRs) and 95% confidence intervals (CIs) were calculated.

Given the limited number of recurrence events, the final multivariable model was deliberately restricted to a small number of prespecified clinically relevant covariates (sex, AJCC stage, and BRAF V600E mutational status) to reduce the risk of overfitting. The multivariable analysis was performed using a complete-case approach; patients with missing data for any of the covariates included in the final model were excluded, resulting in 82 patients and 29 recurrence events being included in the final model. No imputation of missing data was performed. A formal statistical test of the proportional hazards assumption was not performed because of the limited sample size and number of recurrence events. Therefore, the Cox regression estimates should be interpreted as exploratory associations rather than definitive effect estimates, and the possibility of model instability, residual model misspecification, and overfitting cannot be completely excluded.

Because adjuvant anti-PD-1 therapy was introduced during the later years of the study period, treatment allocation was largely determined by calendar year rather than by baseline clinicopathological characteristics. Therefore, adjuvant therapy was not included as a covariate in the multivariable Cox regression model. Instead, an exploratory subgroup analysis was performed according to adjuvant treatment status to evaluate whether the association between AJCC stage and recurrence differed between treated and untreated patients. This analysis was not designed to estimate a treatment effect or to establish a predictive interaction between stage and adjuvant therapy. Categorical variables were compared using Pearson’s Chi-square test or Fisher’s exact test, as appropriate.

## 3. Results

### 3.1. Clinicopathological Characteristics and Nodal Microanatomy

A total of 97 patients with stage IIB-IIIB cutaneous melanoma were included in the study. The baseline histopathological, clinical, and molecular characteristics of the study population are detailed in Table 1. The median age at diagnosis was 60.3 years (standard deviation (SD) = 15.3) and 58.8% of patients were younger than 65 years. The cohort comprised 53 men (54.6%) and 44 women (45.4%). Regarding disease stage at diagnosis, 21 patients (21.6%) were classified as stage IIB, 21 (21.6%) as stage IIC, 20 (20.6%) as stage IIIA and 35 (36.1%) as stage IIIB.

Histologically, 53 patients (54.6%) presented with lower-risk melanoma subtypes, including superficial spreading melanoma and other favorable variants, whereas 44 patients (45.4%) harbored higher-risk variants. SLNB was positive in 47 (48.5%) patients and negative in 45 (46.4%), while status was unknown in 5 patients. Of the 83 patients with available molecular data, BRAF V600E mutation was identified in 38 cases (39.2% of the total cohort).

In the sub-cohort of 47 patients with positive nodal disease (SLN+), detailed microanatomical and tumor burden evaluations were performed. According to the Dewar topographic classification, metastatic deposits within the sentinel lymph node were categorized as subcapsular in 15 patients (31.9%), combined in 5 (10.6%), parenchymal in 3 (6.4%), multifocal in 8 (17.0%), and extensive in 3 (6.4%); data were unavailable for the remaining 13 cases (27.7%). Regarding maximum tumor burden using the Rotterdam criteria, deposit diameters were ≤1 mm in 17 patients (36.2%), >1 to ≤5 mm in 11 (23.4%), and >5 mm in 6 (12.8%). Cellular morphology was categorized as aggregates in 19 patients (40.4%), isolated tumor cells in 14 (29.8%), and nodular deposits in 3 (6.4%).

### 3.2. Statistical Comparisons and Univariate Analysis of Recurrence and Recurrence-Free Survival (RFS)

After a median follow-up of 88 months, 31 patients (32.0%) developed disease recurrence, with a median time to recurrence of 14 months. Regional or distant metastases constituted the majority of recurrences (83.9%, *n* = 26), while isolated local recurrence occurred in only five patients (16.1%). The median OS was 68.9 months (95% CI: 39.0–309.0). The estimated mean RFS for the entire cohort was 122.2 months (95% CI: 105.7–138.8), and the median RFS was not reached.

Associations between clinicopathological factors and recurrence were assessed using contingency table analyses, whereas recurrence-free survival was evaluated using Kaplan–Meier curves and compared with the log-rank test (Table 2).

Patient sex revealed a statistically significant association with RFS (log-rank *p* = 0.014), wherein female patients exhibited a significantly longer mean period free of recurrence [141.72 months (95% CI: 120.90–162.54)] compared to male patients [104.11 months (95% CI: 80.08–128.15)]. In categorical models, recurrence occurred in 41.5% of male patients compared with 20.5% of female patients (Pearson’s Chi-square *p* = 0.027).

AJCC clinical stage was also significantly associated with a decline in RFS (log-rank *p* = 0.026). Stage IIC was associated with the most unfavorable prognosis, recording a mean RFS of 73.97 months, a median RFS of 20.00 months (95% CI: 4.22–35.78), and the highest recurrence rate across the cohort (52.4%). In contrast, the median RFS was not reached for stages IIB, IIIA, and IIIB, which demonstrated prolonged mean survival times of 137.63, 138.08, and 116.23 months, respectively, with stage IIIB recording a recurrence rate of 31.4%. No direct statistical comparison between stages IIC and IIIB was performed; therefore, these numerical differences should not be interpreted as strong evidence that stage IIC has worse outcomes than stage IIIB. Within the tumor microenvironment, a higher density of lymphoid infiltration demonstrated a highly significant impact on delaying disease recurrence (log-rank *p* = 0.006); this finding was considered exploratory because assessment was missing in 47 patients and was not quantitatively standardized.

Regarding molecular profile, BRAF V600E status demonstrated a distinct clinical trend toward accelerated recurrence, reaching borderline statistical significance in categorical association (44.7% recurrence in mutated vs. 26.7% in wild-type; *p* = 0.066) and a trend in the RFS distribution curves (log-rank *p* = 0.103). Patients with wild-type BRAF presented a favorable prognosis, with a mean RFS of 132.22 months, while the presence of a BRAF V600E mutation reduced the mean RFS to 87.71 months, with a median RFS reached at 82.00 months. These univariable findings should be interpreted as exploratory and do not establish an independent prognostic effect of BRAF V600E.

The distribution of BRAF V600E mutations also varied across AJCC stage groups. Among patients with available BRAF status, BRAF V600E mutations were identified in 4/18 patients (22.2%) with stage IIB, 9/17 (52.9%) with stage IIC, 4/14 (28.6%) with stage IIIA, and 21/34 (61.8%) with stage IIIB disease. This distribution suggests an uneven representation of BRAF V600E-mutated tumors across stage groups, with the highest proportion observed in stage IIIB and a relatively high proportion also observed in stage IIC. However, given the limited sample size and incomplete availability of molecular data, this finding should be considered exploratory. Whether differences in BRAF V600E distribution contribute to the clinical heterogeneity observed across AJCC stage groups requires confirmation in larger cohorts.

Within the positive sentinel lymph node sub-cohort (*n* = 47), detailed exploratory analysis of microanatomical features revealed no statistically significant correlations with survival outcomes or recurrence rates. According to the Dewar topographic classification (log-rank *p* = 0.524), the median RFS was 59.0 months for subcapsular, 97.0 months for combined, 55.0 months for parenchymal, 27.0 months for multifocal, and 82.0 months for extensive involvement. Increasing maximum tumor burden according to the Rotterdam criteria was not significantly associated with worse RFS (log-rank *p* = 0.588), with median RFS recorded at 68.0 months for the ≤1 mm group, 28.0 months for the >1–5 mm group, and 55.0 months for the >5 mm group. No significant differences were observed for metastatic morphology subgroups (log-rank *p* = 0.569). Traditional markers—including melanoma subtype, tumor ulceration, mitotic index, lymphovascular invasion, and the execution of a completion lymphadenectomy—were not significantly associated with RFS in univariable analyses (all *p* ≥ 0.05).

### 3.3. Exploratory Analysis According to Adjuvant Treatment

Since adjuvant anti-PD-1 therapy became available during the study period, an exploratory subgroup analysis was performed according to adjuvant treatment status. This analysis was intended to describe stage-associated recurrence patterns within treatment strata and was not designed to estimate the efficacy of adjuvant therapy or to test a treatment-by-stage interaction.

Among the 33 patients who received adjuvant therapy, recurrence rates did not differ significantly according to AJCC stage (Fisher–Freeman–Halton exact test, *p* = 0.386). However, interpretation of this exploratory analysis is limited by the small number of patients with stage IIB (*n* = 3) and stage IIC disease (*n* = 3).

**Figure 1:**
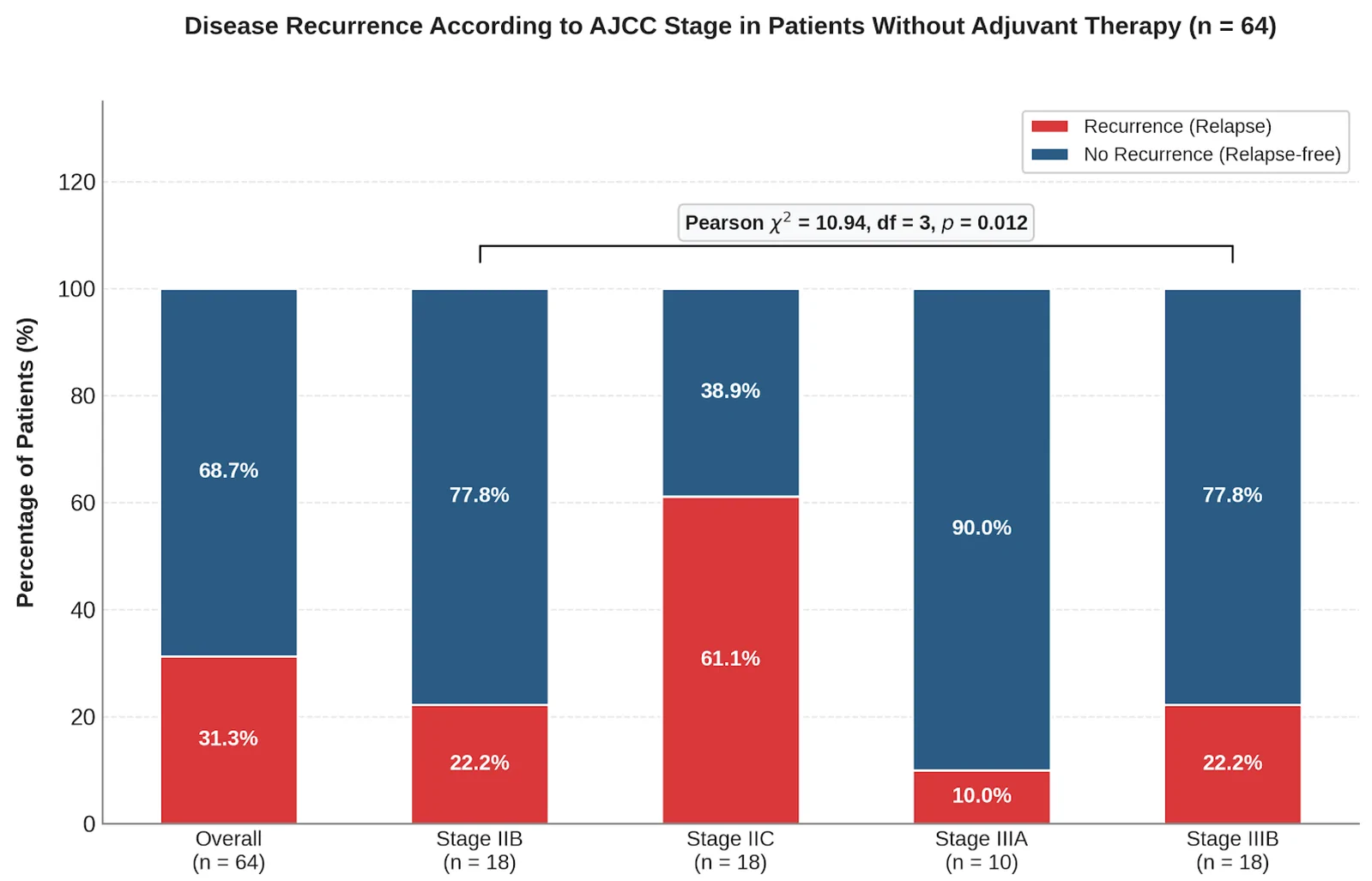
Recurrence rates according to AJCC stage among patients who did not receive adjuvant therapy.

Conversely, among the 64 patients who did not receive adjuvant therapy, AJCC stage was significantly associated with recurrence (Pearson’s χ^2^ = 10.94, degrees of freedom (df) = 3, *p* = 0.012) with stage IIC showing the highest recurrence rate (61.1%) (Figure 1, Table 3). These subgroup findings should be regarded as descriptive and hypothesis-generating rather than as evidence of a treatment effect.

**Table 3:** Association between AJCC stage and recurrence among patients who did not receive adjuvant therapy (*p* = 0.012).

AJCC 8th Edition Stage	Total Patients, *n* (%)	Recurrence (Relapse), *n* (%)	No Recurrence (Relapse-Free), *n* (%)	Statistical Significance
**All Patients (Untreated Cohort)**	**64 (100.0%)**	**20 (31.3%)**	**44 (68.8%)**	**—**
Stage IIB	18 (28.1%)	4 (22.2%)	14 (77.8%)	Ref.
**Stage IIC**	**18 (28.1%)**	**11 (61.1%)**	**7 (38.9%)**	***p*** = 0.012 *
Stage IIIA	10 (15.6%)	1 (10.0%)	9 (90.0%)	—
Stage IIIB	18 (28.1%)	4 (22.2%)	14 (77.8%)	—

Categorical variables were compared using Pearson’s Chi-square test (Chi-square = 10.94, df = 3, *p* = 0.012). * Statistically significant difference (*p* < 0.05). Note the marked increase in relapse rate for stage IIC compared to stage IIB and stage III subgroups.

### 3.4. Multivariate Cox Regression Analysis

**Figure 2:**
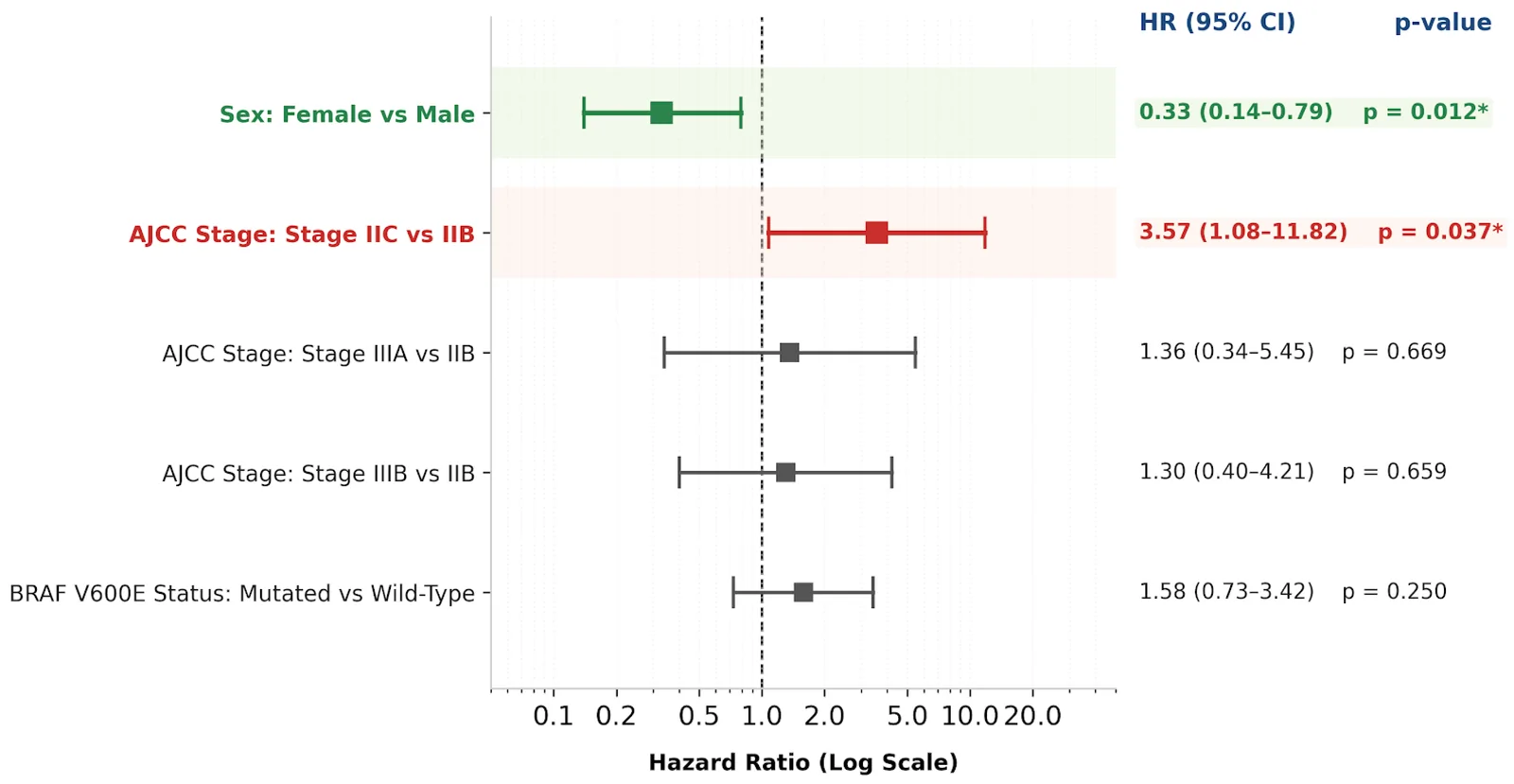
Forest plot of multivariate Cox regression analysis showing hazard ratios (HRs) and 95% confidence intervals (CIs) for recurrence-free survival (RFS). The vertical line indicates HR = 1, and the x-axis is displayed on a logarithmic scale. * *p* < 0.05.

A multivariable Cox proportional hazards regression analysis was performed to evaluate the association between selected clinicopathological variables and RFS. Based on clinical relevance and considering the limited number of recurrence events, the final model included sex, stages, and BRAF V600E status. After exclusion of patients with missing covariate data, 82 patients, including 29 recurrence events, were included in the multivariable analysis. The results are summarized in Figure 2. The multivariable model was fitted using complete-case analysis. The model therefore included five regression parameters for 29 events (5.8 events per parameter). Although the number of covariates was deliberately restricted, the low event count and absence of a formal proportional hazards test mean that the model should be regarded as exploratory and potentially susceptible to instability or overfitting. The multivariable model was fitted using complete-case analysis.

In the multivariable model, female sex remained significantly associated with improved RFS (HR = 0.33, 95% CI 0.14–0.79; *p* = 0.012). Compared with stage IIB, stage IIC was associated with an increased risk of recurrence (HR = 3.57, 95% CI 1.08–11.82; *p* = 0.037), whereas no significant differences were observed for stage IIIA (HR = 1.36, 95% CI 0.34–5.45; *p* = 0.669) or stage IIIB (HR = 1.30, 95% CI 0.40–4.21; *p* = 0.659). BRAF V600E status was not significantly associated with RFS (HR = 1.58, 95% CI 0.73–3.42; *p* = 0.250). Given the limited number of recurrence events, the complete-case selection, the relatively wide confidence intervals, and the absence of formal testing of the proportional hazards assumption, these estimates should be interpreted as exploratory associations rather than definitive effect estimates. The model was intentionally parsimonious to reduce the risk of overfitting; nevertheless, residual model instability and potential violations of model assumptions cannot be excluded.

## 4. Discussion

This retrospective study evaluated a real-world cohort of patients with high-risk cutaneous melanoma (stages IIB–IIIB) with a median follow-up of 88 months. The principal findings were that female sex and stage IIC remained independently associated with recurrence-free survival after multivariable adjustment in our cohort, whereas higher lymphoid infiltration density was associated with improved RFS in univariable survival analysis. These findings suggest clinically relevant heterogeneity of high-risk melanoma [15,16]. Given the small sample size, limited number of recurrence events, missing data, and small subgroups available for detailed pathological and treatment analyses, the results should be considered hypothesis-generating rather than definitive.

In our cohort, male patients experienced a significantly higher recurrence rate than female patients (41.5% vs. 20.5%, *p* = 0.027) and demonstrated a markedly shorter recurrence-free survival (RFS) (104.11 months for males vs. 141.72 months for females, *p* = 0.014). These results are consistent with previous epidemiological studies reporting a persistent survival disadvantage in men with melanoma, even after adjusting for established prognostic factors [7,8]. Although the underlying mechanisms remain incompletely understood, several explanations have been proposed. These include differences in tumor presentation, health-seeking behavior, hormonal influences, and sex-related immune responses [7,8]. Importantly, our multivariable Cox regression analysis confirmed that female sex was independently associated with a lower risk of recurrence (HR = 0.33, 95% CI: 0.14–0.79; *p* = 0.012). These data support the potential role of sex as a prognostic factor, although these findings should be interpreted cautiously given the sample size [23].

A major point of clinical debate in contemporary oncology centers is the accuracy of the American Joint Committee on Cancer (AJCC) staging system for predicting long-term outcomes in high-risk disease [7]. Although regional lymph node involvement has traditionally been regarded as the main determinant of poor prognosis, stage IIC showed the highest numerical recurrence rate in our cohort (52.4%), compared with 31.4% in stage IIIB. This observation is consistent with population-based evidence suggesting that the risk of recurrence in stage IIC may be comparable to that observed in stage IIIB disease [24]. Stage IIC also showed the shortest median RFS (20.00 months) and was independently associated with an increased risk of recurrence compared with stage IIB (HR = 3.57, 95% CI: 1.08–11.82; *p* = 0.037), whereas no significant differences were observed for stage IIIA or IIIB. Given the limited sample size and number of recurrence events, these findings should be interpreted as exploratory and warrant validation in larger, independent cohorts.

To explore whether the observed recurrence pattern according to AJCC stage might have been influenced by the use of adjuvant therapy, we performed a descriptive subgroup analysis according to adjuvant treatment status. Among patients who did not receive adjuvant therapy, AJCC stage remained significantly associated with recurrence (Pearson’s χ^2^ = 10.94, *p* = 0.012), with stage IIC showing the highest recurrence rate (61.1%), compared with 22.2% in both stages IIB and IIIB. Conversely, no significant association between stage and recurrence was observed among patients treated with adjuvant therapy (Fisher–Freeman–Halton exact test, *p* = 0.386). However, these analyses were not designed to evaluate treatment efficacy or treatment-by-stage interactions, and the number of treated patients with stage IIB and IIC disease was very small (*n* = 3 in each group). Therefore, these findings should be considered descriptive and hypothesis-generating.

Regarding the sub-analysis of patients with a positive sentinel lymph node (*n* = 47), lymphoid infiltration density was significantly associated with recurrence (*p* = 0.006). This finding raises the possibility that the local immune microenvironment may contribute to differences in recurrence risk, although this hypothesis requires confirmation using standardized pathological and immune assessments in larger cohorts [25,26].

These findings may have clinical relevance in the context of recent randomized trials such as KEYNOTE-716 and CheckMate 76K, which demonstrated a benefit of adjuvant anti-PD-1 therapy in resected stage IIB–IIC melanoma and have led to changes in treatment guidelines [17,18,19]. Our results further support the need for refined risk stratification within established AJCC stage groups, particularly among patients with high-risk stage II disease.

An additional biological consideration is the potential role of therapy-persistent melanoma cells within minimal residual disease. Experimental studies have shown that a subset of melanoma cells can survive therapeutic pressure in reversible, non-genetic persister states characterized by slow proliferation and phenotypic plasticity. These cells may subsequently contribute to tumor repopulation and disease recurrence [27]. In resected melanoma, the concept of minimal residual disease is particularly relevant because clinically undetectable residual tumor cells may persist after surgery and may be detected through molecular approaches such as circulating tumor DNA (ctDNA) analysis [28,29,30]. Recent studies in high-risk stage II/III melanoma have demonstrated the feasibility of serial ctDNA monitoring and suggested an association between postoperative ctDNA detection and recurrence risk [28,29]. Moreover, longitudinal ctDNA monitoring in patients with stage I–IIIB melanoma, including substantial cohorts of patients with stage IIB and IIC disease, has shown a strong association between postoperative ctDNA positivity and shorter recurrence-free survival [29,30]. Although our retrospective study did not include molecular residual disease assessment or serial ctDNA analysis, this biological framework may provide a potential explanation for why patients with apparently localized disease, including stage IIC melanoma, may experience recurrence despite complete surgical resection. Future studies incorporating ctDNA and other markers of minimal residual disease could therefore complement conventional AJCC staging and help identify patients at highest risk of recurrence.

A fundamental objective of our study was to evaluate whether the detailed microanatomical characteristics of SLN provided additional prognostic information beyond conventional staging. Historically, the Rotterdam tumor load criteria and the Dewar topographic classification have been associated with survival and the risk of non-sentinel node involvement [21,22]. In our exploratory univariate analysis, however, none of these advanced microanatomical features correlated significantly with recurrence-free survival, including the Dewar classification (log-rank *p* = 0.524), Rotterdam tumor burden (log-rank *p* = 0.588), and metastatic cell morphology (log-rank *p* = 0.569). These findings should be interpreted cautiously given the limited number of patients with available SLN microanatomical data (*n* = 47) and the substantial proportion of missing assessments for some pathological variables. Therefore, the prognostic relevance of these features could not be demonstrated in this cohort and requires further evaluation in larger series using standardized pathological assessments.

At the molecular level, BRAF V600E mutational status showed a trend toward an association with recurrence in univariable analysis, but it did not retain independent prognostic significance in the multivariable model, which is consistent with the known biological role of BRAF-driven MAPK pathway activation in melanoma progression [20]. Patients harboring a BRAF V600E mutation showed shorter RFS in our cohort, although these findings should be interpreted cautiously given the limited sample size and incomplete molecular data. Clinical evidence suggests that BRAF V600E-mutated tumors have been associated with distinct biological features and patterns of disease dissemination. However, these tumors also display distinct molecular characteristics that influence their response to targeted therapy and immune checkpoint inhibition [20]. Further studies in larger cohorts are needed to clarify the prognostic and predictive role of BRAF status in this setting [20]. The uneven distribution of BRAF V600E mutations across AJCC stage groups may also contribute to differences in recurrence patterns and warrants further investigation in larger cohorts.

Interestingly, traditional histopathological variables including melanoma subtype, ulceration, mitotic index, lymphovascular invasion, and completion lymph node dissection were not independently associated with recurrence-free survival in our cohort. These findings are consistent with the current shift toward less invasive management following the MSLT-II and DeCOG-SLT trials, which questioned the prognostic benefit of completion lymph node dissection [11,12].

We acknowledge several limitations of this study. The retrospective single-center design and relatively small sample size limit the generalizability of the findings. The limited number of recurrence events and complete-case approach may have reduced the statistical stability of the multivariable model and increased the risk of overfitting; therefore, the multivariable results should be considered exploratory and require external validation. Incomplete availability of molecular and pathological variables may also have introduced selection bias, particularly for BRAF V600E status and lymphoid infiltration. In addition, the small numbers within treatment subgroups and the introduction of adjuvant therapy during the later study period limited the interpretation of treatment-stratified analyses. Finally, because no direct statistical comparison between stage IIC and IIIB was performed, the numerical difference in recurrence rates between these groups should not be interpreted as evidence of superior or inferior outcomes. Nevertheless, the long median follow-up of 88 months represents an important strength of the study, allowing mature assessment of recurrence patterns in a real-world cohort.

## 5. Conclusions

In conclusion, female sex was associated with improved recurrence-free survival whereas stage IIC was associated with poorer recurrence-free survival in this exploratory cohort of patients with high-risk cutaneous melanoma. However, these findings should be interpreted cautiously given the small sample size, limited number of recurrence events, and incomplete availability of some clinicopathological and molecular variables. Importantly, our findings do not challenge the validity of the AJCC staging system but suggest that clinically relevant heterogeneity may exist within established AJCC stage groups and warrants further investigation in larger cohorts. The prognostic associations observed for BRAF V600E status and lymphoid infiltration, as well as the analyses according to adjuvant treatment, should be considered exploratory and hypothesis-generating. Further prospective studies incorporating standardized pathological, molecular, and immune biomarkers are needed to determine whether these factors can improve risk stratification within established AJCC stages.

## Figures and Tables

**Table 1:** Baseline clinicopathological and SLN+ characteristics of the cohort (*n* = 97).

Variable	Total Cohort (*n* = 97)	Percentage (%)
**Sex**		
Female	44	45.4%
Male	53	54.6%
**Mean Age (years)**	60.29	SD ± 15.30
**Age Distribution**		
<65 years	57	58.8%
≥65 years	40	41.2%
**AJCC 8th Edition Stage**		
IIB	21	21.6%
IIC	21	21.6%
IIIA	20	20.6%
IIIB	35	36.1%
**Anatomical Location**		
Trunk/head and neck	55	56.7%
Extremities	42	43.3%
**Histological Risk Profile**		
Lower risk (superficial spreading/favorable variants)	53	54.6%
Higher risk (nodular/acral lentiginous/pure desmoplastic)	44	45.4%
**Sentinel Lymph Node Biopsy (SLNB)**		
Positive	47	48.5%
Negative	45	46.4%
Unknown	5	5.2%
**Completion Lymphadenectomy**		
Yes	30	30.9%
No	67	69.1%
**Ulceration Status**		
Present	30	30.9%
Absent	67	69.1%
**Mitotic Index**		
Present	85	87.6%
Absent	9	9.3%
Unknown	3	3.1%
**Lymphovascular Invasion**		
Yes	4	4.1%
No	93	95.9%
**Histological Regression**		
Yes	9	9.3%
No	88	90.7%
**Lymphoid Infiltration**		
Mild	29	29.9%
Moderate	12	12.4%
Severe	3	3.1%
Absent	5	5.2%
Mild–moderate	1	1.0%
Unknown	47	48.5%
**BRAF V600E Status**		
Wild-Type (WT)	45	46.4%
Mutated	38	39.2%
Unknown	14	14.4%
**Recurrence Status**		
Recurrence	31	32.0%
Recurrence-free	66	68.0%
**Positive SLN Microanatomy Subgroup (*n* = 47)**		
**Dewar Topographic Classification**		
Subcapsular	15	31.9%
Combined	5	10.6%
Parenchymal	3	6.4%
Multifocal	8	17.0%
Extensive	3	6.4%
Unknown	13	27.7%
**Rotterdam Tumor Load (Diameter)**		
≤1 mm	17	36.2%
>1 to ≤5 mm	11	23.4%
>5 mm	6	12.8%
Unknown	13	27.7%
**Metastatic Cell Morphology**		
Aggregates	19	40.4%
Isolated tumor cells	14	29.8%
Nodular deposits	3	6.4%
Unknown	11	23.4%

**Table 2:** Association of clinicopathological factors with recurrence and recurrence-free survival.

Variable/Subgroup	Total *n* (%)	Recurrence: Yes (*n*/%)	Recurrence: No (*n*/%)	Categorical *p*-Value	Mean RFS (Months, 95% CI)	Median RFS (Months, 95% CI)	Log-Rank *p*-Value
**All Patients**	97 (100)	31 (32.0)	66 (68.0)	—	122.20 (105.7–138.8)	NR	—
**Sex**							
Female	44 (45.4)	9 (20.5)	35 (79.5)	0.027 *	141.72 (120.9–162.5)	NR	0.014 *
Male	53 (54.6)	22 (41.5)	31 (58.5)		104.11 (80.1–128.2)	NR	
**AJCC Clinical Stage**							
Stage IIB	21 (21.6)	4 (19.0)	17 (81.0)	**0.107**	137.63 (108.6–166.7)	NR	0.026 *
Stage IIC	21 (21.6)	11 (52.4)	10 (47.6)		73.97 (44.9–103.0)	20.00 (4.2–35.8)	
Stage IIIA	20 (20.6)	5 (25.0)	15 (75.0)		138.08 (107.5–168.7)	NR	
Stage IIIB	35 (36.1)	11 (31.4)	24 (68.6)		116.23 (92.5–139.9)	NR	
**Melanoma Subtype Profile**							
Lower-risk variants	53 (54.6)	17 (32.1)	36 (67.9)	**0.978**	117.84 (96.8–138.9)	NR	**0.974**
Higher-risk variants	44 (45.4)	14 (31.8)	30 (68.2)		121.71 (96.3–147.1)	NR	
**Sentinel Node Biopsy**							
Positive	47 (48.5)	15 (31.9)	32 (68.1)	**0.832**	105.15 (82.1–128.2)	NR	**0.805**
Negative	45 (46.4)	15 (33.3)	30 (66.7)		129.42 (106.6–152.2)	NR	
Unknown	5 (5.2)	1 (20.0)	4 (80.0)		—	—	
**Completion Lymphadenectomy**							
Yes	30 (30.9)	12 (40.0)	18 (60.0)	**0.256**	97.43 (68.9–126.0)	NR	**0.483**
No	67 (69.1)	19 (28.4)	48 (71.6)		129.21 (110.1–148.3)	NR	
**Primary Tumor Ulceration**							
Present	30 (30.9)	10 (33.3)	20 (66.7)	**0.846**	115.12 (86.1–144.1)	NR	**0.721**
Absent	67 (69.1)	21 (31.3)	46 (68.7)		123.90 (104.3–143.5)	NR	
**Mitotic Index**							
Present	85 (87.6)	27 (31.8)	58 (68.2)	**0.994**	121.45 (103.7–139.2)	NR	**0.989**
Absent	9 (9.3)	3 (33.3)	6 (66.7)		114.22 (64.8–163.6)	NR	
Unknown	3 (3.1)	1 (33.3)	2 (66.7)		—	—	
**Lymphovascular Invasion**							
Yes	4 (4.1)	2 (50.0)	2 (50.0)	**0.429**	54.50 (1.4–107.6)	48.00 (0.0–116.3)	**0.324**
No	93 (95.9)	29 (31.2)	64 (68.8)		124.62 (107.9–141.4)	NR	
**Histological Regression**							
Yes	9 (9.3)	2 (22.2)	7 (77.8)	**0.511**	134.11 (86.2–182.0)	NR	**0.375**
No	88 (90.7)	29 (33.0)	59 (67.0)		119.51 (101.9–137.1)	NR	
**Anatomical Location**							
Trunk/head and neck	55 (56.7)	20 (36.4)	35 (33.6)	**0.287**	113.84 (93.1–134.6)	NR	**0.263**
Extremities	42 (43.3)	11 (26.2)	31 (73.8)		130.65 (106.6–154.7)	NR	
***BRAF*** **Status**							
Mutated	38 (39.2)	17 (44.7)	21 (55.3)	**0.066**	87.71 (64.3–111.1)	82.00 (NR)	**0.103**
Wild-Type (WT)	45 (46.4)	12 (26.7)	33 (73.3)		132.22 (111.7–152.8)	NR	
Unknown	14 (14.4)	2 (14.3)	12 (85.7)		—	—	
**Lymphoid Infiltration Density**	50 (100)	—	—	**0.476**	—	—	0.006 *
**Positive SLN Subgroup (*n* = 47)**							
Dewar Topographic Class							
Subcapsular	15 (31.9)	7 (46.7)	8 (53.3)	**0.604**	61.13 (35.0–87.3)	59.00 (12.3–105.7)	**0.524**
Combined	5 (10.6)	2 (40.0)	3 (60.0)		79.20 (21.5–137.0)	97.00 (0.0–279.5)	
Parenchymal	3 (6.4)	1 (33.3)	2 (66.7)		68.67 (22.0–115.3)	55.00 (24.6–85.4)	
Multifocal	8 (17.0)	1 (12.5)	7 (87.5)		42.50 (20.1–64.9)	27.00 (0.0–65.8)	
Extensive	3 (6.4)	1 (33.3)	2 (66.7)		87.67 (16.7–158.6)	82.00 (0.0–168.4)	
Rotterdam Diameter							
≤1 mm	17 (36.2)	4 (23.5)	13 (76.5)	**0.781**	77.53 (49.1–106.0)	68.00 (4.8–131.2)	**0.588**
>1 to ≤5 mm	11 (23.4)	2 (18.2)	9 (81.8)		56.55 (26.1–87.0)	28.00 (0.0–70.1)	
>5 mm	6 (12.8)	2 (33.3)	4 (66.7)		68.50 (30.5–106.5)	55.00 (31.0–79.0)	
Metastatic Morphology							
Aggregates	19 (40.4)	6 (31.6)	13 (68.4)	**0.471**	—	—	**0.569**
Isolated cells	14 (29.8)	5 (35.7)	9 (64.3)		—	—	
Nodular deposits	3 (6.4)	0 (0.0)	3 (100)		—	—	

Categorical variables were compared using the Chi-square test or Fisher’s exact test, as appropriate. Recurrence-free survival was compared using the log-rank test. * Statistically significant value (*p* ≤ 0.05). NR: Not reached. —: Estimates could not be calculated due to a low number of events or small sample sizes within specific sub-categories.

## Data Availability

The data presented in this study are not publicly available due to privacy and ethical restrictions concerning patient confidentiality. All patient data were anonymized prior to analysis, and no directly identifiable patient information was included in the study. The underlying data may be available from the corresponding author upon reasonable request, subject to applicable ethical, legal, and privacy restrictions.

## Abbreviations

The following abbreviations are used in this manuscript:

RFS	Recurrence-free survival
SLN	Sentinel lymph node
SLNB	Sentinel lymph node biopsy
AJCC	American Joint Committee on Cancer
ECOG	Eastern Cooperative Oncology Group
OS	Overall survival
HR	Hazard ratio
CI	Confidence interval
